# Efficacy of vitamin D supplementation in depression in adults: a systematic review protocol

**DOI:** 10.1186/2046-4053-2-64

**Published:** 2013-08-08

**Authors:** Guowei Li, Lawrence Mbuagbaw, Zainab Samaan, Shiyuan Zhang, Jonathan D Adachi, Alexandra Papaioannou, Lehana Thabane

**Affiliations:** 1 Department of Clinical Epidemiology & Biostatistics, McMaster University, 1280 Main Street West, Hamilton, ON L8S 4L8, Canada; 2 Department of Psychiatry and Behavioural Neurosciences, McMaster University, 1280 Main Street West, Hamilton, ON L8S 4L8, Canada; 3 St. Joseph’s Hospital, McMaster University, 25 Charlton Avenue East, Hamilton, ON L8N 1Y2, Canada; 4 Division of Geriatric Medicine, Department of Medicine, McMaster University, 1280 Main Street West, Hamilton, ON L8S 4L8, Canada

**Keywords:** Vitamin D, Depression, Randomized controlled trial, Adults, Efficacy, Systematic review protocol

## Abstract

**Background:**

The role of vitamin D in management of depression is unclear. Results from observational and emerging randomized controlled trials (RCTs) investigating the efficacy of vitamin D in depression lack consistency - with some suggesting a positive association while others show a negative or inconclusive association.

**Methods/Design:**

The primary aim of this study is to conduct a systematic review of RCTs to assess the effect of oral vitamin D supplementation versus placebo on depression symptoms measured by scales and the proportion of patients with symptomatic improvement according to the authors’ original definition. Secondary aims include assessing the change in quality of life, adverse events and treatment discontinuation. We will conduct the systematic review and meta-analysis according to the recommendations of the Cochrane Handbook for Systematic Reviews of Interventions. We will search the Cochrane Central Register of Controlled Trials (CENTRAL), MEDLINE (1966 to present), EMBASE (1980 to present), CINAHL (1982 to present), PsychINFO (1967 to present) and ClinicalTrials.gov. Unpublished work will be identified by searching two major conferences: the International Vitamin Conference, the Anxiety Disorders and Depression Conference, while grey literature will be acquired by contacting authors of included studies. We will use the random-effects meta-analysis to synthesize the data by pooling the results of included studies.

**Discussion:**

The results of this systematic review will be helpful in clarifying the efficacy of vitamin D supplementation and providing evidence to establish guidelines for implementation of vitamin D for depression in general practice and other relevant settings.

**Study registration:**

Unique identifier: CRD42013003849.

## Background

Depression is characterized by a depressed mood or loss of interest or pleasure in almost all daily activities for a period of at least two weeks [1]. Depression is the fourth leading cause of an increase in disability-adjusted life-years (DALY) worldwide [2], and it is projected to be the second leading cause of burden of disease by 2030 after human immunodeficiency virus (HIV) and Acquired Immune Deficiency Syndrome (AIDS) [3].

The Diagnostic and Statistical Manual of Mental Disorders (DSM) IV has well-defined operational criteria for diagnosing depressive disorders for trained clinicians. However many other screening and research diagnostic tools are also available, including the *Feighner Criteria*[4], *Research Diagnostic Criteria*[5], self-rating questionnaires (such as *Beck Depression Inventory* (BDI) [6,7]*, Center for Epidemiologic Studies Depression scale* (CES-D) [8]*, General Health Questionnaire* (GHQ) [9]*,* etcetera) and clinician-rating scales (such as *Hamilton Depression Rating Scale* (HAM-D) [10,11]*, Montgomery-Åsberg Depression Rating Scale* (MADRS) [12]*, Inventory for Depressive Symptomatology-Clinician Rated or Self-rated* (IDS-C/SR) [13], etcetera). Clinical practice guidelines for the treatment of depression recommend the use of antidepressants, cognitive-behavior therapy, and interpersonal psychotherapy [14]. Nevertheless, patients are prone to fail to receive optimal treatment due to its poor public acceptability [15] and their variable response to treatment. More simple and acceptable interventions are urgently needed.

Vitamin D is produced endogenously in the skin by sun exposure, and humans also obtain vitamin D from the diet and from supplements to a minor extent. The recommended intake of vitamin D varies from 200 international units (IU) to 1000 IU per day [16]. Vitamin D supplementation increases serum 25-hydroxy vitamin D (25(OH)D) levels, thereby potentially correcting the effects of vitamin D deficiency [16]. Both vitamin D2 (ergocalciferol) and vitamin D3 (cholecalciferol) are available as supplementations to maintain serum 25(OH)D concentrations. However, since vitamin D3 is considered to be more potent than vitamin D2 [17,18], vitamin D3 supplementation has been widely used with different doses in trials related to depression [19-22].

It is commonly known that vitamin D is essential for the maintenance of calcium homeostasis and for bone health [16], but its role in the brain is not fully understood. Because the receptor of vitamin D is found in areas of the brain that are involved in the development of depression [23], vitamin D and its relationship to depressive symptoms and other psychiatric disorders are under investigation [24,25]. Treatment of depression with vitamin D has potentially profound implications, because for patients in whom vitamin D is an effective antidepressant, it will be one of the most cost-effective treatments in psychiatry, and one with negligible side effects [26].

A number of observational studies have investigated the relationship between depression and vitamin D with conflicting results. While cross-sectional studies identified an association between low level of serum 25(OH)D and scores on measures of depression inconclusively [27-30], some prospective studies [31-33] using large samples reported a significant association. However, it is difficult to corroborate the causality in observational research due to the numerous potential confounders including age, time spent outdoors, latitude, physical activity, body mass index, smoking, alcohol use, etcetera [34]. Emerging randomized controlled trials (RCTs) of vitamin D supplementation have been reported, but their findings were also inconsistent. For instance, Jorde *et al*. [35] found an effect of high dose vitamin D supplementation on depressive symptoms, whereas other research failed to observe a significant treatment effect [20,36]. Therefore, it is essential to summarize the best available evidence to date to clarify the efficacy of vitamin D.

A recent systematic review investigating the relationship between vitamin D deficiency and depression in adults presented significant positive associations in observational studies [37]. Nevertheless, it did not include any RCT up to February 2011 according to the authors’ criterion, thereby failing to assess the efficacy of vitamin D supplementation in depression in trials. Plenty of RCTs on vitamin D and depression have been published since then. Thus, to identify the efficacy of vitamin D supplementation in depression in adults with depressive symptoms/diagnosis or at risk of depression, we will conduct a comprehensive systematic review and meta-analysis of RCTs.

### Aims

The overall purpose of this systematic review is to evaluate the efficacy of oral vitamin D supplementation in depression in adults with depressive symptoms/diagnosis or at risk of depression in RCTs. The primary aim is to assess the effect of oral vitamin D supplementation versus placebo on depression symptoms measured by scales (for continuous outcome) and the proportion of patients with symptomatic improvement according to the authors’ original definition (for dichotomous outcome). Secondary aims include assessing the change in quality of life, adverse events and treatment discontinuation.

## Methods/Design

### Criteria for considering studies for this review

#### Type of studies

We will include only RCTs investigating the effect of oral vitamin D supplementation on depression in adults (18 years of age and over). Only conventional parallel designs will be eligible, while cross-over RCTs will be excluded because depressive symptoms may not be static and participants’ variability is hard to interpret.

#### Type of participants

We will include adults who are at risk of depression, have depressive symptoms, or have a primary diagnosis of depression based on the authors’ definition. Risk factors for depression in our study are manipulated as: having a family history of depression [38], obesity for adults [39], postpartum period for women [40], perimenopause for women in midlife [41], bereavement, sleep disturbance, disability, prior depression and female gender for the elderly [42]. Subjects with a diagnosed depressive disorder or with depressive symptoms that are secondary to another medical condition will be included, but trials in which the primary focus is another major psychiatric condition such as anxiety disorders, will be excluded. Studies involving participants with vitamin D abnormalities such as hyperparathyroidism, will also be excluded. If the same participants are assessed in different time points or in multiple studies, we will extract and analyze all the data of different follow-up periods, and choose those with the largest sample size of the same follow-up period for analysis.

#### Type of interventions

At least one of the intervention arms has to include oral intake of vitamin D as a mono-intervention. We will consider all doses and durations of vitamin D supplementation. Studies that combined vitamin D with any other vitamin, antidepressant, calcium, or light therapy will be excluded, because we want to isolate the intervention effect due to vitamin D and obtain its efficacy by direct comparison with placebo.

#### Comparison

Only trials using placebos in their control groups will be included. Specifically, the comparison will be oral vitamin D supplementation versus placebo.

#### Type of outcome measures

##### Primary outcome

Our primary outcome is the effect of oral vitamin D supplementation on improvement in the depression symptoms measured by depression symptoms scales (continuous outcome) and the proportion of patients with symptomatic improvement according to the authors’ definition (dichotomous outcome), compared to placebo. If the original authors report outcomes using several different scales corresponding with our definition of response, we will give preference to BDI for self-rating questionnaires and HAM-D for observer-rating scales.

##### Secondary outcome

Secondary outcomes include:

1. change in quality of life;

2. adverse events; and

3. treatment discontinuation.

### Search strategy

#### Electronic searching

We will search the Cochrane Central Register of Controlled Trials (CENTRAL), MEDLINE (1966 to present), EMBASE (1980 to present), CINAHL (1982 to present), PsychINFO (1967 to present) and ClinicalTrials.gov exhaustively and comprehensively. In our searches, we will use descriptors that include synonyms for depression, vitamin D and randomized controlled trials in various combinations, for example, 'vitamin D or 25 hydroxyvitamin D' and 'depression or mood disorder' and 'RCT or clinical trial'. We will first search MEDLINE (see Table 1). Subsequent search strategies will be derived from the MEDLINE strategy and adapted for each database. Our searches will not be limited by language, publication status or setting.

**Table 1:** MEDLINE search terms

**Outcome**	**Descriptor**
Search for vitamin D supplementation	1. Vitamin D/explode
	2. vitamin D
	3. vitamin D2
	4. vitamin D3
	5. 1-alpha hydroxyvitamin D3
	6. 1-alpha-hydroxy-vitamin D3
	7. 1-alpha hydroxycalciferol
	8. 1-alpha-hydroxy-calciferol
	9. 1,25 dihydroxyvitamin D3
	10. 1,25-dihydroxy-vitamin D3
	11. 1,25 dihydroxycholecalciferol
	12. 1,25-dihydroxycholecalciferol
	13. 25-hydroxycholecalciferol
	14. 25 hydroxycholecalciferol
	15. 25 hydroxyvitamin D
	16. 25-hydroxy-vitamin D
	17. alfacalcidol
	18. calcidiol
	19. calcitriol
	20. calcifediol
	21. calciferol
	22. ergocalciferol
	23. cholecalciferol
	24. OR/1-23
Search for depression	25. Depress*
	26. Dysthymi*
	27. Adjustment Disorder*
	28. Mood Disorder*
	29. Affective Disorder
	30. Affective Symptoms
	31. OR/25-30
Search for randomized controlled trials	32. trial
	33. clinical trial
	34. random* controlled trial
	35. RCT
	36. group*
	37. experiment*
	38. intervention
	39. placebo
	40. OR/32-39
Search for combinations	41. 24 AND 31 AND 40

#### Reference lists

The reference lists of articles and other reviews retrieved in the search or known to the authors will be searched for relevant articles.

#### Conference abstracts

Unpublished work will be identified by searching the abstract books or websites of two major conferences: the International Vitamin Conference, the Anxiety Disorders and Depression Conference. Any abstract of interest will be assessed for further detail by contacting the authors.

#### Grey literature

We will try to contact authors of included studies to acquire other data that may either be unpublished or informally published or ongoing and which is related to efficacy of vitamin D in depression.

### Data collection and analysis

A summary of the identification, screening and inclusion of studies in this review will be presented as a PRISMA (Preferred Reporting Items for Systematic Reviews and Meta-analyses) diagram [43].

#### Selection of studies

Two review authors (GL and SZ) will independently screen and select studies for possible inclusion in the study. First, the titles and abstracts of trials identified from the search will be independently reviewed and pooled for further screening. Secondly, each review author will independently examine the full text of all trials that were identified from the title and abstract screens. Each reviewer will compile a list of studies that meet the inclusion criteria. The contents of each review author’s list will be compared, and any disagreement will be resolved by discussion and consensus between all of the review authors. Agreement between authors will be quantified using the Kappa statistic [44].

#### Data extraction and management

Two review authors (GL and SZ) will independently extract data using specially developed data extraction forms. Information will be collected on:

1. participant characteristics (age, sex, numbers of participants, diagnosis or symptoms of depression, co-morbidity, severity of depression, study setting including season and latitude where study was conducted, inclusion and exclusion criteria in the included studies, baseline serum 25(OH)D and the assay method, washout periods for antidepressants and other supplements);

2. intervention details (number of arms, sample size for each, randomization and allocation concealment method, blinding, dose and type of supplementation, duration, withdrawals, and drop-outs);

3. outcome measures (description of measures used, continuous/dichotomous nature, results of intervention including scores of depression and interim/final serum 25(OH)D, and adverse outcomes).

We will pilot the data extraction form prior to its use. Any disagreement will be resolved by discussion and consensus between all of the review authors.

#### Statistical analysis

A random-effects meta-analysis will be performed throughout to synthesize the data by pooling the results of the included studies. Heterogeneity between included studies will be assessed using both the chi-square test and the I^2^ statistic [45,46]. In addition, to make the probability statement for the efficacy of vitamin D and to incorporate the prior beliefs and external information (that is, observational data), we will synthesize the results from the RCTs using a hierarchical Bayesian random-effects model [47-49] in conjunction with observational studies included in a recent systematic review [37]. Specifically, observational studies investigating relationship between vitamin D measurements as a risk factor and depression as the outcome of interest in adults will be eligible for pooled analysis as prior distributions to conduct Bayesian meta-analysis.

We will analyze the data using Review Manager (RevMan) version 5.2 for windows (the Nordic Cochrane Center, the Cochrane Collaboration, Copenhagen, Denmark). We will present the results with 95% confidence intervals. We will calculate the pooled risk ratio (RR) and the odds ratio (OR) for dichotomous data, the weighted mean difference (WMD) for continuous data measured on the same scale, and the standardized mean difference (SMD) for data measured on different scales [50].

We will use the software WinBUGS 1.4 (MRC Biostatistics Unit, Cambridge, UK) to apply three prior distributions to the Bayesian random-effects model: a 'non-informative' or 'vague' prior distribution [51,52], an 'informative' prior distribution [47,53] and a 'skeptical' prior distribution [52], the latter two of which will be based on the pooled observational studies [37]. The efficacy of the intervention will then be acquired from the posterior distribution of the Bayesian analysis, presenting as a SMD, or a RR (or OR) with 95% associated credible interval.

### Dealing with missing data

For missing or unclear data, the authors of the studies will be contacted during eligibility assessment and data abstraction. Missing data will also be sought from secondary publications of the same study. However, if data are only available in graphic format, we will impute approximations of the mean. If the effort to seek further information from original authors or secondary studies is fruitless, in order to estimate standard deviations (SDs) we will borrow SDs from other trials included in this meta-analysis [54].

### Assessment of risk of bias in included studies

We will assess risk of bias for each included study by an adapted Cochrane Collaboration 'Risk of bias' assessment tool [50], including sequence generation, allocation concealment, blinding, incomplete outcome data/loss to follow-up, selective outcome reporting and other issues.

The response options for the quality assessment are defined as: yes (criteria applied and described appropriately or acknowledged in the study), no (criteria inappropriately applied) and unclear (criteria not described and impossible to obtain from the study). Each study will then be classified into one of the categories below.

• High risk of bias: one or more criteria not applied/met.

• Moderate risk of bias: one or more criteria unclear.

• Low risk of bias: all criteria applied/met.

The review authors will discuss any disagreement in the assessment of risk of bias to reach a consensus.

### Assessment of quality of evidence across studies

We will assess the quality of evidence in this systematic review using the Grading of Recommendations Assessment, Development and Evaluation (GRADE) tool [55] with GRADEprofiler (GRADEpro) version 3.6 software, defining the quality of evidence for each outcome as the extent to which one can be confident that an estimate of effect or relation is close to the quantity of specific interest [50]. The GRADE system rates the quality of evidence across studies as one of four levels: very low, low, moderate and high. RCTs are categorized as high quality but can be downgraded for several reasons, including limitation in study design, indirectness of evidence, imprecision of results, unexplained heterogeneity or inconsistency of results, or high probability of publication bias [55].

### Assessment of heterogeneity in included studies

We will first assess clinical heterogeneity by determining whether the studies are similar enough to pool. In the event that they are, statistical heterogeneity will be evaluated using the I^2^ statistic, with a value of I^2^ >50% or *P* value <0.1 taken as implying significant heterogeneity [45,46]. If statistical heterogeneity is found, it will be examined by subgroup and sensitivity analyses.

#### Subgroup analysis

We plan to investigate the heterogeneity by carrying out the following subgroup analyses:

1. different vitamin D dosages (that is, less than 4,000 IU/day versus more than 4,000 IU/day);

2. different study settings (that is, high versus low latitude where study was conducted);

3. males versus females;

4. institutional versus community dwellers; and

5. clinical versus general population sample.

#### Sensitivity analysis

We hypothesize that vitamin D supplementation will be less effective on depression in studies with a high risk of bias and in studies with short duration (that is, less than six months), thus we will carry out sensitivity analyses by excluding studies classified as having high risk of bias and removing those having short duration. Also, a fixed-effects model will be conducted for sensitivity analysis.

### Assessment of reporting biases

We will construct a funnel plot to investigate the potential for publication bias for the primary outcomes relating to the diagnosis or symptoms of depression, by means of visual inspection for signs of asymmetry, Begg’s rank correlation and Egger’s regression tests [50] using the STATA metabias command.

## Discussion

The efficacy of vitamin D supplementation in depression has raised lots of concern. Vitamin D is considered as a neurosteroid [56], and now it is attested that vitamin D metabolites can cross the blood–brain barrier [34]. Because of the widespread presence of vitamin D receptor in areas of the brain including the hippocampus which is associated with the development of depression [23], it could be speculated that there is a clinical effect of vitamin D on depression.

The initial suggestion that vitamin D may be linked to depression was based on the relation between low vitamin D and high prevalence of seasonal affective disorder in winter at high latitudes [57]. Since then a number of observational studies had been published, but they yielded inconclusive results mainly as to whether the lower levels of vitamin D were a cause or consequence of depression [26,34]. Also several RCTs had looked at the efficacy of vitamin D supplements in depression, and if any, the association between depression and the positive effect by vitamin D supplementation that may indicate a causative relation. But, the findings of these trials were not uniform [20,35,36,58]. Divergent results have been reported in various population, study settings, duration, etcetera.

A review by Bertone-Johnson concluded that the evidence linking vitamin D to the development of depression remains largely circumstantial, after analyzing several observational studies and one RCT [34]. Two other reviews also demonstrated that it was premature to conclude a significant clinical effect of vitamin D supplementation on depression [59,60], while Anglin *et al*. failed to summarize the evidence of RCTs [37]. Therefore, a systematic review based on RCTs will yield a better understanding of the efficacy of vitamin D in depression, which will be helpful in establishing guidelines for implementation in general practice and other relevant settings.

To our knowledge, this systematic review and meta-analysis is the first to evaluate the efficacy of vitamin D supplementation in depression in RCTs. Summarizing the available RCT evidence on the efficacy of vitamin D will be very useful, because there is a positive public perception of oral vitamin D supplementation, which could lead to high rates of patient adherence [61]. Furthermore, vitamin D supplementation may be cost-effective with rare adverse effects [16] in preventing development of depression or treating depressive symptoms.

We anticipate that the review will provide valuable evidence of beneficial efficacy of vitamin D supplementation in depression. The review will also probably inform clinicians and healthcare providers about a simple and acceptable intervention and method that will serve the needs of people at risk of depression or with depressive symptoms, or depression patients.

## Abbreviations

AIDS: Acquired Immune Deficiency Syndrome; BDI: Beck Depression Inventory; CES-D: Center for Epidemiologic Studies Depression scale; DALY: Disability-adjusted life-years; DSM: Diagnostic and Statistical Manual of Mental Disorders; GHQ: General Health Questionnaire; GRADE: Grading of Recommendations Assessment Development and Evaluation; HAM-D: Hamilton Depression Rating Scale; HIV: Human immunodeficiency virus; IDS-C/SR: Inventory for Depressive Symptomatology-Clinician Rated or Self-rated; MADRS: Montgomery-Åsberg Depression Rating Scale; OR: Odds ratio; RCT: Randomized controlled trial; RR: Risk ratio; SD: Standard deviation; SMD: Standardized mean difference; WMD: Weighted mean difference; 25(OH)D: 25-hydroxyvitamin D.

## Competing interests

The authors declare that they have no competing interests.

## Authors’ contributions

GL and LT were responsible for the study conception and design. GL, LM and SZ were responsible for the drafting of the manuscript. LM, ZS, JA, AP and LT made several critical revisions and provided professional and statistical support. All authors read and approved the final manuscript.

## References

[B1] American Psychiatric Association Diagnostic and Statistical Manual of Mental Disorders, Fourth Edition, Text Revision (DSM-IV-TR) 2000 Washington, DC: American Psychiatric Association

[B2] Murray CJ Lopez AD Global mortality, disability, and the contribution of risk factors: Global Burden of Disease Study Lancet 1997 349 1436 1442 10.1016/S0140-6736(96)07495-8 9164317

[B3] Mathers CD Loncar D Projections of global mortality and burden of disease from 2002 to 2030 PLoS Med 2006 3 e442 10.1371/journal.pmed.0030442 17132052 PMC1664601

[B4] Feighner JP Robins E Guze SB Woodruff RA Jr Winokur G Munoz R Diagnostic criteria for use in psychiatric research Arch Gen Psychiatry 1972 26 57 63 10.1001/archpsyc.1972.01750190059011 5009428

[B5] Spitzer RL Robins E Endicott J Research Diagnostic Criteria (RDC) for a Selected Group of Functional Disorders 1978 3 New York, NY: New York State Psychiatric Institute

[B6] Beck AT Ward CH Mendelson M Mock J Erbaugh J An inventory for measuring depression Arch Gen Psychiatry 1961 4 561 571 10.1001/archpsyc.1961.01710120031004 13688369

[B7] Beck AT Steer RA Brown GK BDI-II, Beck Depression Inventory: Manual 1996 2 San Antonio: Texas: Psychological Corporation

[B8] Radloff LS The CES-D scale: A self-report depression scale for research in the general population Appl Psychol Meas 1977 1 385 401 10.1177/014662167700100306

[B9] Goldberg D The Detection of Psychiatric Illness by Questionnaire 1972 Oxford: Oxford University Press

[B10] Hamilton M A rating scale for depression J Neurol Neurosurg Psychiatry 1960 23 56 62 10.1136/jnnp.23.1.56 14399272 PMC495331

[B11] Hamilton M Development of a rating scale for primary depressive illness Br J Soc Clin Psychol 1967 6 278 296 10.1111/j.2044-8260.1967.tb00530.x 6080235

[B12] Montgomery SA Asberg M A new depression scale designed to be sensitive to change Br J Psychiatry 1979 134 382 389 10.1192/bjp.134.4.382 444788

[B13] Rush AJ Giles DE Schlesser MA Fulton CL Weissenburger J Burns C The Inventory for Depressive Symptomatology (IDS): preliminary findings Psychiatry Res 1986 18 65 87 10.1016/0165-1781(86)90060-0 3737788

[B14] Ellis P Australian and New Zealand clinical practice guidelines for the treatment of depression Aust N Z J Psychiatry 2004 38 389 407 15209830 10.1080/j.1440-1614.2004.01377.x

[B15] Jorm AF Mental health literacy. Public knowledge and beliefs about mental disorders Br J Psychiatry 2000 177 396 401 10.1192/bjp.177.5.396 11059991

[B16] Holick MF Vitamin D deficiency N Engl J Med 2007 357 266 281 10.1056/NEJMra070553 17634462

[B17] Armas LA Hollis BW Heaney RP Vitamin D2 is much less effective than vitamin D3 in humans J Clin Endocrinol Metab 2004 89 5387 5391 10.1210/jc.2004-0360 15531486

[B18] Houghton LA Vieth R The case against ergocalciferol (vitamin D2) as a vitamin supplement Am J Clin Nutr 2006 84 694 697 17023693 10.1093/ajcn/84.4.694

[B19] Sanders KM Stuart AL Williamson EJ Jacka FN Dodd S Nicholson G Berk M Annual high-dose vitamin D3 and mental well-being: randomised controlled trial Br J Psychiatry 2011 198 357 364 10.1192/bjp.bp.110.087544 21525520

[B20] Kjaergaard M Waterloo K Wang CE Almas B Figenschau Y Hutchinson MS Svartberg J Jorde R Effect of vitamin D supplement on depression scores in people with low levels of serum 25-hydroxyvitamin D: nested case–control study and randomised clinical trial Br J Psychiatry 2012 201 360 368 10.1192/bjp.bp.111.104349 22790678

[B21] Dean AJ Bellgrove MA Hall T Phan WM Eyles DW Kvaskoff D McGrath JJ Effects of vitamin D supplementation on cognitive and emotional functioning in young adults–a randomised controlled trial PLoS One 2011 6 e25966 10.1371/journal.pone.0025966 22073146 PMC3208539

[B22] Bertone-Johnson ER Powers SI Spangler L Larson J Michael YL Millen AE Bueche MN Salmoirago-Blotcher E Wassertheil-Smoller S Brunner RL Ockene I Ockene JK Liu S Manson JE Vitamin D supplementation and depression in the women’s health initiative calcium and vitamin D trial Am J Epidemiol 2012 176 1 13 10.1093/aje/kwr482 22573431 PMC3385159

[B23] Eyles DW Smith S Kinobe R Hewison M McGrath JJ Distribution of the vitamin D receptor and 1 alpha-hydroxylase in human brain J Chem Neuroanat 2005 29 21 30 10.1016/j.jchemneu.2004.08.006 15589699

[B24] Hoogendijk WJ Lips P Dik MG Deeg DJ Beekman AT Penninx BW Depression is associated with decreased 25-hydroxyvitamin D and increased parathyroid hormone levels in older adults Arch Gen Psychiatry 2008 65 508 512 10.1001/archpsyc.65.5.508 18458202

[B25] Armstrong DJ Meenagh GK Bickle I Lee AS Curran ES Finch MB Vitamin D deficiency is associated with anxiety and depression in fibromyalgia Clin Rheumatol 2007 26 551 554 10.1007/s10067-006-0348-5 16850115

[B26] Young SN Has the time come for clinical trials on the antidepressant effect of vitamin D? J Psychiatry Neurosci 2009 34 3 19125208 PMC2612077

[B27] Hoang MT Defina LF Willis BL Leonard DS Weiner MF Brown ES Association between low serum 25-hydroxyvitamin D and depression in a large sample of healthy adults: the Cooper Center longitudinal study Mayo Clin Proc 2011 86 1050 1055 10.4065/mcp.2011.0208 22033249 PMC3202994

[B28] Jorde R Waterloo K Saleh F Haug E Svartberg J Neuropsychological function in relation to serum parathyroid hormone and serum 25-hydroxyvitamin D levels. The Tromso study J Neurol 2006 253 464 470 10.1007/s00415-005-0027-5 16283099

[B29] Zhao G Ford ES Li C Balluz LS No associations between serum concentrations of 25-hydroxyvitamin D and parathyroid hormone and depression among US adults Br J Nutr 2010 104 1696 1702 10.1017/S0007114510002588 20642877

[B30] Nanri A Mizoue T Matsushita Y Poudel-Tandukar K Sato M Ohta M Mishima N Association between serum 25-hydroxyvitamin D and depressive symptoms in Japanese: analysis by survey season Eur J Clin Nutr 2009 63 1444 1447 10.1038/ejcn.2009.96 19690578

[B31] Tolppanen AM Sayers A Fraser WD Lewis G Zammit S Lawlor DA The association of serum 25-hydroxyvitamin D3 and D2 with depressive symptoms in childhood–a prospective cohort study J Child Psychol Psychiatry 2012 53 757 766 10.1111/j.1469-7610.2011.02518.x 22211693 PMC3412227

[B32] Milaneschi Y Shardell M Corsi AM Vazzana R Bandinelli S Guralnik JM Ferrucci L Serum 25-hydroxyvitamin D and depressive symptoms in older women and men J Clin Endocrinol Metab 2010 95 3225 3233 10.1210/jc.2010-0347 20444911 PMC2928895

[B33] Bertone-Johnson ER Powers SI Spangler L Brunner RL Michael YL Larson JC Millen AE Bueche MN Salmoirago-Blotcher E Liu S Wassertheil-Smoller S Ockene JK Ockene I Manson JE Vitamin D intake from foods and supplements and depressive symptoms in a diverse population of older women Am J Clin Nutr 2011 94 1104 1112 10.3945/ajcn.111.017384 21865327 PMC3173027

[B34] Bertone-Johnson ER Vitamin D and the occurrence of depression: causal association or circumstantial evidence? Nutr Rev 2009 67 481 492 10.1111/j.1753-4887.2009.00220.x 19674344 PMC2950608

[B35] Jorde R Sneve M Figenschau Y Svartberg J Waterloo K Effects of vitamin D supplementation on symptoms of depression in overweight and obese subjects: randomized double blind trial J Intern Med 2008 264 599 609 10.1111/j.1365-2796.2008.02008.x 18793245

[B36] Vieth R Kimball S Hu A Walfish PG Randomized comparison of the effects of the vitamin D3 adequate intake versus 100 mcg (4000 IU) per day on biochemical responses and the wellbeing of patients Nutr J 2004 3 8 10.1186/1475-2891-3-8 15260882 PMC506781

[B37] Anglin RE Samaan Z Walter SD McDonald SD Vitamin D deficiency and depression in adults: systematic review and meta-analysis Br J Psychiatry 2013 202 100 107 10.1192/bjp.bp.111.106666 23377209

[B38] Grayson L Thomas A A systematic review comparing clinical features in early age at onset and late age at onset late-life depression J Affect Disord 2013 13 00246 2 10.1016/j.jad 23623421

[B39] Luppino FS de-Wit LM Bouvy PF Stijnen T Cuijpers P Penninx BW Zitman FG Overweight, obesity, and depression: a systematic review and meta-analysis of longitudinal studies Arch Gen Psychiatry 2010 67 220 229 10.1001/archgenpsychiatry.2010.2 20194822

[B40] O’hara MW Swain AM Rates and risk of postpartum depression-a meta-analysis Int Rev Psychiatry 1996 8 37 54 10.3109/09540269609037816

[B41] Cohen LS Soares CN Vitonis AF Otto MW Harlow BL Risk for new onset of depression during the menopausal transition: the Harvard study of moods and cycles Arch Gen Psychiatry 2006 63 385 390 10.1001/archpsyc.63.4.385 16585467

[B42] Cole MG Dendukuri N Risk factors for depression among elderly community subjects: a systematic review and meta-analysis Am J Psychiatry 2003 160 1147 1156 10.1176/appi.ajp.160.6.1147 12777274

[B43] Moher D Liberati A Tetzlaff J Altman DG Preferred reporting items for systematic reviews and meta-analyses: the PRISMA statement BMJ 2009 339 b2535 10.1136/bmj.b2535 19622551 PMC2714657

[B44] Viera AJ Garrett JM Understanding interobserver agreement: the kappa statistic Fam Med 2005 37 360 363 15883903

[B45] Higgins JP Thompson SG Quantifying heterogeneity in a meta-analysis Stat Med 2002 21 1539 1558 10.1002/sim.1186 12111919

[B46] Higgins JP Thompson SG Deeks JJ Altman DG Measuring inconsistency in meta-analyses BMJ 2003 327 557 560 10.1136/bmj.327.7414.557 12958120 PMC192859

[B47] Sutton AJ Abrams KR Bayesian methods in meta-analysis and evidence synthesis Stat Methods Med Res 2001 10 277 303 10.1191/096228001678227794 11491414

[B48] Smith TC Spiegelhalter DJ Thomas A Bayesian approaches to random-effects meta-analysis: a comparative study Stat Med 1995 14 2685 2699 10.1002/sim.4780142408 8619108

[B49] Sung L Hayden J Greenberg ML Koren G Feldman BM Tomlinson GA Seven items were identified for inclusion when reporting a Bayesian analysis of a clinical study J Clin Epidemiol 2005 58 261 268 10.1016/j.jclinepi.2004.08.010 15718115

[B50] Higgins JPT Green S Cochrane Handbook for Systematic Reviews of Interventions 5.1.0 http://handbook.cochrane.org

[B51] Lambert PC Sutton AJ Burton PR Abrams KR Jones DR How vague is vague? A simulation study of the impact of the use of vague prior distributions in MCMC using WinBUGS Stat Med 2005 24 2401 2428 10.1002/sim.2112 16015676

[B52] Spiegelhalter DJ Abrams KR Myles JP Bayesian approaches to clinical trials and health care evaluation Statistics in Practice; Variation: Statistics in Practice 2004 Hoboken, NJ: John Wiley & Sons 139 180

[B53] Higgins JP Whitehead A Borrowing strength from external trials in a meta-analysis Stat Med 1996 15 2733 2749 10.1002/(SICI)1097-0258(19961230)15:24<2733::AID-SIM562>3.0.CO;2-0 8981683

[B54] Furukawa TA Barbui C Cipriani A Brambilla P Watanabe N Imputing missing standard deviations in meta-analyses can provide accurate results J Clin Epidemiol 2006 59 7 10 10.1016/j.jclinepi.2005.06.006 16360555

[B55] Guyatt GH Oxman AD Vist GE Kunz R Falck-Ytter Y Alonso-Coello P Schunemann HJ GRADE: an emerging consensus on rating quality of evidence and strength of recommendations BMJ 2008 336 924 926 10.1136/bmj.39489.470347.AD 18436948 PMC2335261

[B56] Garcion E Wion-Barbot N Montero-Menei CN Berger F Wion D New clues about vitamin D functions in the nervous system Trends Endocrinol Metab 2002 13 100 105 10.1016/S1043-2760(01)00547-1 11893522

[B57] Stumpf WE Privette TH Light, vitamin D and psychiatry. Role of 1,25 dihydroxyvitamin D3 (soltriol) in etiology and therapy of seasonal affective disorder and other mental processes Psychopharmacology (Berl) 1989 97 285 294 10.1007/BF00439440 2497477

[B58] Lansdowne AT Provost SC Vitamin D3 enhances mood in healthy subjects during winter Psychopharmacology (Berl) 1998 135 319 323 10.1007/s002130050517 9539254

[B59] Eyles DW Burne TH McGrath JJ Vitamin D, effects on brain development, adult brain function and the links between low levels of vitamin D and neuropsychiatric disease Front Neuroendocrinol 2013 34 47 64 10.1016/j.yfrne.2012.07.001 22796576

[B60] McCann JC Ames BN Is there convincing biological or behavioral evidence linking vitamin D deficiency to brain dysfunction? FASEB J 2008 22 982 1001 18056830 10.1096/fj.07-9326rev

[B61] Straube S Derry S Moore RA McQuay HJ Vitamin D for the treatment of chronic painful conditions in adults Cochrane Database Syst Rev 2010 1 CD007771 20091647 10.1002/14651858.CD007771.pub2 PMC4170888

